# From suspicion of cognitive decline to dementia diagnosis: a systematic review of healthcare professionals’ considerations and attitudes

**DOI:** 10.1093/ageing/afaf176

**Published:** 2025-06-24

**Authors:** Fleur C W Visser, Marleen Kloppenburg-Lagendijk, Liesbeth Hempenius, Nicolaas A Verwey, Marieke Perry, Marlise E A van Eersel, Barbara C van Munster

**Affiliations:** Department of Geriatric Medicine, University Medical Center Groningen, University of Groningen, Groningen, The Netherlands; Alzheimer Center Groningen, University Medical Center Groningen, University of Groningen, Groningen, The Netherlands; Department of Geriatric Medicine, Medical Center Leeuwarden, Leeuwarden, The Netherlands; Department of Geriatric Medicine, Medical Center Leeuwarden, Leeuwarden, The Netherlands; Neurology and Geriatric Department, Medical Center Leeuwarden, Leeuwarden, The Netherlands; Vrije Universiteit, Amsterdam UMC, Neuroscience Campus, Amsterdam, The Netherlands; Department of Primary and Community Care, Radboud University Medical Center, Radboudumc Alzheimer Center, Nijmegen, The Netherlands; Radboud University Medical Center, Radboudumc Alzheimer Center, Nijmegen, The Netherlands; Department of Geriatric Medicine, University Medical Center Groningen, University of Groningen, Groningen, The Netherlands; Alzheimer Center Groningen, University Medical Center Groningen, University of Groningen, Groningen, The Netherlands; Department of Geriatric Medicine, University Medical Center Groningen, University of Groningen, Groningen, The Netherlands; Alzheimer Center Groningen, University Medical Center Groningen, University of Groningen, Groningen, The Netherlands; Department of Geriatric Medicine, Martini Hospital, Groningen, The Netherlands

**Keywords:** diagnosis, Alzheimer’s disease, timely diagnosis, healthcare professionals, primary care, decision-making, considerations, attitudes, qualitative research, older people

## Abstract

**Background:**

Initiating diagnostic testing for dementia is a dynamic and complex process that often involves balancing competing interests. This systematic review aims to provide an overview of healthcare professionals’ considerations and attitudes during the process from suspicion of cognitive decline to deciding to initiate diagnostic testing.

**Methods:**

Databases (PubMed, EMBASE, CINAHL and PsychINFO) were systematically searched on 29 January 2024 for qualitative and mixed-methods studies published since 2005. Search concepts were: ‘dementia’, ‘considerations and attitudes’, ‘healthcare professionals’ and ‘diagnosis’. Two screeners independently conducted title/abstract-screening using ASReview (efficient and transparent systematic review machine learning framework), and full-text screening. Findings were analysed by thematic synthesis.

**Results:**

Thirty-three studies were included. Most involved primary care physicians (*n* = 25), primary care nurses (*n* = 1) or a combination (*n* = 7). The overarching phenomenon was that starting the diagnostic workup for dementia is a delicate process. Clusters influencing this process were: complexities arising from the nature of dementia; interaction with the patient and family; individual determinants of primary care practitioners (PCPs); expectations regarding the consequences of a diagnosis; factors related to the healthcare system; and societal factors. Together these clusters form PCPs’ strategies and actions for deciding whether to start the diagnostic workup.

**Conclusion:**

Initiating the diagnostic workup for dementia is a delicate process influenced by various factors including fear, reluctance and stigma. The different strategies that PCPs use cannot be captured by a single right approach. Recommendations to better support PCPs in navigating this complex process include ensuring consistent communication and clarity about their roles, and promoting interprofessional collaboration.

## Key Points

- Initiating the diagnostic workup for dementia is a delicate process influenced by various factors.
- Some of the complexities include fear, stigma and reluctance, and complicate a shared decision with patients and families.
- The different strategies that primary care practitioners (PCPs) use to address the complexities cannot be captured by a single right approach.
- Recommendations are ensuring consistent communication, clarity regarding their roles and promoting interprofessional collaboration.
- PCPs’ confidence could be enhanced, particularly in managing conflicting priorities and interpreting cognitive test results.

## Introduction

The growing number of individuals with dementia poses challenges in providing appropriate care [1]. The first step in providing appropriate care is to make a timely diagnosis. ‘Timely’ means diagnosing dementia at a moment that best aligns with the person’s preferences and individual situation, without necessarily indicating a specific stage of the disease [2]. Thus, shared decision-making is important to determine the most suitable moment for the well-being of the person with dementia and their family members [3]. A dementia diagnosis may offer clarity regarding the cause of cognitive decline, access to care, measures to slow decline and the opportunity for future planning. However, it can also lead to emotional distress, an inability for some patients to comprehend the diagnosis, and practical challenges, such as the potential loss of a driving licence [4, 5].

There is still much to improve in the timeliness of dementia diagnosis [6–9]. To achieve a timely dementia diagnosis, an explicit shared decision should be made about starting diagnostic testing between the person with suspected dementia, their family and their healthcare professional (HCP) [3]. However, patients and family members regularly felt they did not have an explicit choice and, accordingly, were not involved in shared decision-making [9]. A survey conducted by Alzheimer Europe reported that more than half of the family members stated an earlier diagnosis would have been preferable [6]. In addition, a systematic review showed that over 60% of individuals with dementia are undetected [7].

HCPs play a central role in the decision-making process for a dementia diagnosis. General practitioners (GPs) or nurse practitioners are usually the first HCPs to approach for concerns related to dementia. Additionally, due to shared risk factors of chronic illnesses and dementia, individuals with symptoms of dementia regularly encounter HCPs [10–12]. This interaction places HCPs in a position to observe and address cognitive decline, particularly when individuals do not seek help themselves [13, 14]. Moreover, due to their medical training, HCPs typically should have a better understanding of dementia than most relatives or the general public.

Research on HCPs’ experiences shows that initiating diagnostic testing for dementia is a dynamic and complex process that often involves balancing competing interests [11, 15]. Better understanding HCPs’ considerations and attitudes in their interaction with patients and families may reveal opportunities to improve the timeliness of dementia diagnosis. Therefore, this systemic review aims to provide an overview of the considerations and attitudes of HCPs during the process from suspicion of cognitive decline to deciding to initiate diagnostic testing.

## Methods

This review was performed according to the preferred reporting items for systematic reviews and meta-analysis (PRISMA) guidelines [16]. The protocol was registered prospectively with the Open Science Framework (https://doi.org/10.17605/OSF.IO/RSC89).

### Search strategy and selection criteria

PubMed, EMBASE, CINAHL and PsychINFO were systematically searched to identify relevant articles. The search terms were defined using Mesh-terms and related free text terms for each concept: ‘dementia’, ‘healthcare professionals’, ‘considerations and attitudes’ and ‘diagnosis’. The search strategy was developed and modified for each database with equivalent index terms in consultation with a research librarian (see Appendix 1 in the Supplementary Data for the PubMed string). The search was limited to studies published in English, Dutch and German since 2005. This timeframe was chosen because two articles on expert group evaluations from that year highlighted the importance of timely recognition and diagnosis [17, 18]. Cited references in eligible studies were searched by hand. The search was conducted on 29 January 2024.

Studies were selected based on inclusion- and exclusion criteria in Table 1. After removing duplicates in Endnote, two reviewers (F.V. and M.K.) conducted title/abstract screening using ASReview software version 1.5 [19, 20]. This machine learning software arranges articles based on initial input of reference papers and continually refines the order during including and excluding items with relevance positioned at the top. ASReview is designed to enhance efficiency in systematic reviews while minimising errors, making it valuable for exploratory research questions where broad and less specific search terms are applicable [19]. To improve the review process’s consistency and calibrate ASReview, the researchers compared their assessments of the initial 50 records. ASReview arranged the remaining records by relevance. Each reviewer continued screening independently until reaching the stop-strategy of 100 consecutive irrelevant records. During the full-text screening process, the two reviewers independently assessed the selected records for eligibility. After both title/abstract screening and full-text screening, the reviewers compared their results. In case of disagreement, a third reviewer (L.H.) was consulted.

**Table 1:** Inclusion and exclusion criteria.

**Inclusion criteria**
Healthcare professionals in primary or hospital care who can initiate the diagnostic process of dementia: primary care physicians, general practitioners, family doctors, practice nurses and medical specialists Studies investigating considerations and attitudes during the process from becoming aware of dementia signs to the decision to initiate diagnostic testing Studies in the primary care or outpatient hospital care setting Peer-reviewed empirical qualitative or mixed-methods studies
**Exclusion criteria**
Studies that only include quantitative evidence Letters, editorials, study protocols, reviews and guidelines Studies that only include information on patient or relative considerations Studies that only include specialist healthcare professionals for whom diagnosing dementia is standard practice (e.g. geriatricians, internist-geriatricians and neurologists) Studies that only include information on healthcare professionals working in inpatient wards Studies only including information on cognitive testing, imaging and referral to memory clinic Studies only reporting on considerations regarding screening for cognitive impairment/dementia in healthy individuals

### Quality assessment

The mixed methods appraisal tool (MMAT) was used to critically evaluate the quality of studies, as it includes assessments for both mixed-methods and qualitative research [21]. Two reviewers (F.V. and M.K.) independently assessed the quality of included studies, discussed their ratings and consulted a third reviewer in case of disagreement. We verified whether the themes identified in studies with a low MMAT score (0%–40% out of 100%) were also present in the moderate to high-quality studies.

### Data extraction and synthesis

Two researchers (F.V. and M.L.) developed the data-extracting form. Characteristics to extract included: author, year of publication, study aim, study design, methodology, data analysis, sample, setting and participants’ professional background, age and gender. One researcher (F.V.) extracted this information and the qualitative findings relevant to the research question. Verbatim qualitative findings of both quotes (first-order constructs) and result sections (second-order constructs) were extracted [22]. A second reviewer (M.K.) validated data-extraction.

We conducted thematic synthesis to integrate the findings using ATLAS.ti [23]. Inductive line-by-line coding was conducted independently by two researchers (F.V. and M.K.) (both having dementia research experience, and F.V. trained and experienced in conducting qualitative research). After coding 15% of articles, the two researchers developed a preliminary coding tree. During an iterative process, researcher 1 (F.V.) coded the remaining articles with this coding tree, and researcher 2 (M.K.) critically reviewed this and applied other codes when the researcher thought something else was more appropriate. In biweekly meetings during this process, the two researchers discussed the discrepancies to refine the codes and adapt the coding tree. In case of remaining discrepancies after discussion among the two coders, a third senior researcher was consulted (L.H.). Subsequently, in several meetings with the other authors (L.H.–PhD, Geriatrician; N.V.–PhD, Neurologist; M.P.–PhD, General Practitioner; M.vE.–PhD, Internist-Geriatrician, B.vM.–PhD, Internist-Geriatrician; M.P. has ample qualitative research experience, the other authors conducted qualitative research before), the preliminary themes and codes were discussed and reformulated if appropriate. We used the coding paradigm developed by Strauss and Corbin during these team discussions to deductively organise the codes on an overarching level [24]. This coding paradigm facilitated identification of an overarching phenomenon across the articles, and ordering of the codes in causes, context, intervening codes, strategies and outcomes. While organising the codes by using this coding paradigm, the team identified the final clusters and slightly modified the paradigm in order to create a better fit for the themes and codes identified. Table 2 shows the final coding tree.

**Table 2:** Identified clusters, themes and codes regarding initiating diagnostic testing for dementia.

*Clusters, themes*	*Codes*	*Sources*
Cluster 1: complexities arising from the nature of dementia		
Diagnosing dementia is complex	The process is complex The process is nuanced and slow to evolve Dementia presents in a dynamic and unpredictable course	[9, 15, 25–34]
The diagnosis has a profound impact	Dementia is a significant diagnosis Essential to get the diagnosis right	[15, 30, 33, 35–41]
Cluster 2: interaction with the patient and family		
Reluctant attitude of patient and/or family	Reluctant to accept or not wanting to know the diagnosis Denial by patient - hide or normalise symptoms, limited insight People do not seek help Challenging to get a patient agree to be tested	[9, 15, 27, 28, 30, 33–36, 39, 40, 42–50]
The patient’s situation and context	The patient’s need, wish and right (not) to know Patient characteristics and symptom severity guide when and how to start diagnostic testing Diagnosing dementia is related to the patient’s context Extensive knowledge of the patient may mitigate against detecting dementia	[9, 15, 27, 33, 35, 39, 41, 43, 46, 47, 50–53]
Fear of dementia	Fear of the diagnosis Diagnosis is emotionally difficult Fear of losing independence and worst-case scenario	[9, 15, 27, 30, 31, 33–41, 43–45, 48, 51, 54]
The availability and attitude of family members	Family members usually report the symptoms History provided by family is essential information Social support system influences decision-making	[9, 15, 25, 32, 33, 35, 37, 38, 40, 42, 46, 49, 50, 54, 55]
Cluster 3: individual determinants of primary care practitioners		
Factors undermining PCPs’ confidence	Lack of knowledge, training or education Not feeling comfortable Complex to interpret results of cognitive tests Difficult to distinguish between dementia and depression or other conditions Difficult to distinguish between normal ageing and cognitive impairment Lack of access to specialist or imaging Contradictions between different information sources	[9, 25–27, 29–36, 42–45, 47–53, 55]
Factors contributing to PCPs’ confidence	PCP feels comfortable conducting cognitive tests and making the diagnosis Confident about diagnosis because of back-up access to specialist Obvious cases of dementia or advanced age	[25, 26, 35, 37, 39, 41]
PCPs’ perceptions of their role	PCPs believe that a diagnosis should be made or confirmed by a specialist The practice nurse may have an important role in identifying and discussing dementia The role of the PCP is recognising cognitive problems and initiating or conducting the diagnostic PCPs have a unique position to act as guide	[15, 26, 30, 31, 33, 34, 37, 41, 44, 47, 48, 50, 52, 55]
PCPs’ perceptions of the aim of the diagnostic process	Meeting patients’ and family members’ needs and preferences Prioritising well-being and safety over confirming a diagnosis Supporting patients to live independently and stay at home	[9, 15, 33, 36, 37, 46, 50, 53, 54]
Cluster 4: expectations of primary care practitioners regarding the consequences of a diagnosis		
Positive (expected) outcomes of diagnosis	Access to care and additional support Future planning and the possibility for patients to decide for themselves Clarity by de-mystifying and naming Counselling or guidance Medication can be used in suitable cases Improving well-being and quality of life Ensuring safety	[9, 15, 25, 27, 31, 35, 36, 39–41, 45, 46, 48, 51, 53, 55]
Limited added value (expected) of diagnosis	No clear effect of treatment No value in early diagnosis Limited sources of care Lack of value in specifying the type of dementia Family and neighbours provide care Patient does not understand diagnosis and implications	[9, 25, 28, 30, 34–38, 40–43, 45, 46, 48, 51, 53, 54]
Negative (expected) outcomes of diagnosis	Diagnosis causes emotional distress Not wanting to medicalise the ageing process Legal and financial issues Driving licence must be taken away	[9, 27, 31, 33, 35–37, 40, 41, 45, 46, 48, 50, 53]
Cluster 5: factors related to the healthcare system		
Failures of dementia policy	Time-constraints in general practice Lack of care available Lack of funding for dementia screening, diagnosis and services Dementia is a low priority health condition	[27, 28, 30, 33–35, 40–45, 47, 48, 50, 51, 54, 55]
Insufficient guidelines and tools	No clear guidelines or referral mechanisms Diagnostic tools provide insufficient guidance Need for availability of an accepted, valid and practical tool for PCPs Diagnostic tools are time-consuming	[25, 27–31, 34, 35, 39, 40, 43, 44, 47, 48, 50, 55]
Advantages of interprofessional collaboration	Strong team culture and interprofessional collaboration facilitates diagnosis Advantages of practice nurse role: extra time, home visit, and less threatening nurse–patient Practice nurses are not always supported as proactive healthcare practitioners	[27, 32, 34, 44, 47, 48, 50]
Cluster 6: societal factors		
Stigma of dementia	Dementia is perceived as a stigma within society Avoidance of the word ‘dementia’ or ‘Alzheimer’s’ in communities Negative attitudes towards people with dementia among HCPs	[9, 25, 26, 30, 31, 33–37, 40, 43–46, 48, 51, 54]
Cultural and language barriers	Dementia is considered a taboo or bad karma Challenges in assessment due to language barriers, illiteracy and non-applicable diagnostic tools	[30, 32, 35, 36, 42, 48–50]
Lack of knowledge and awareness	The belief that memory problems are part of ageing Limited awareness and understanding about dementia	[9, 25, 35, 43, 49, 51, 53–55]
Cluster 7: strategies and actions of primary care practitioners		
Dealing with the challenge of sensitivity	Taking it slowly and gradually introducing the topic Avoidance of the word ‘dementia’ or ‘Alzheimer’s’ in by HCPs Trust enables dealing with the sensitivity PCPs avoid the burden of delivering bad news by referring to the specialist PCPs search for the ‘right’ moment Assessment in home environment provides important information Helping the person rather than focusing on the label PCPs communicate that the patient is in charge during the process PCPs attribute symptoms to another disease as strategy to motivate patients to seek further diagnosis	[9, 15, 30, 33–38, 40, 41, 44–48, 50–52, 54]
Sensitivity causes hesitation among PCPs	Hesitancy to label the patient Difficult how and when to bring up the topic Wait and see approach until problems become evident Fear of ruining the doctor–patient relationship Reactive approach to diagnosis Cognitive test is distressing, embarrassing or uncomfortable	[9, 15, 25, 28, 29, 31–41, 43–48, 50, 51, 53, 54]
PCPs try to weigh up dilemmas	Negotiating conflicting priorities and expectations of involved ones Weighing up benefits and negative consequences of a diagnosis PCPs value and try to apply shared decision-making Balancing patient autonomy and preventing risks or crises	[9, 15, 26, 29, 31–34, 36, 39, 46, 50, 51, 53]
Approaches to start the conversation about dementia	Continuity of care enables recognition and initiation of the conversation Proactive approach, routine health check or screening prompts conversation about dementia Holistic approach supports looking further than the known diseases Having an opening to address concerns Changes picked up during consultations as way to start the conversation	[15, 25, 27, 28, 32, 33, 35, 39, 41, 42, 44, 49–51, 54, 55]

## Results

### Study selection and characteristics

**Figure 1:**
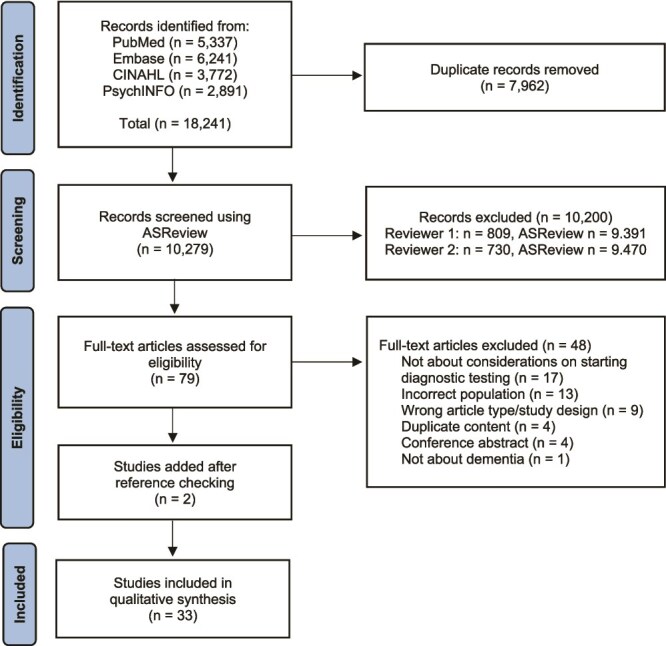
PRISMA flow diagram of study selection.

The search identified 18 241 records. After removing duplicates, 10 279 title/abstracts were screened. This process resulted in 79 articles that were screened in full-text. Cohen’s kappa inter-rater reliability was 0.55 (moderate) and agreement 77% [56]. After full-text screening, 31 articles met the inclusion criteria. Two more articles were identified during reference checking, bringing the total to 33 articles (Figure 1). Table 3 provides an overview of the characteristics of included studies. All studies were conducted in the primary care or community setting, and most were from Europe (*n* = 17) or Australia (*n* = 8). Most study populations involved primary care physicians (*n* = 25), primary care nurses (*n* = 1) or a combination of both (*n* = 7) [9, 15, 25–55].

### Quality assessment

The majority of studies were of moderate to high quality. Sixteen scored 100%, ten scored 80%, five scored 60% and two scored 40%. Individual scores are presented in Table 3 and Supplementary Data Appendix 2. Studies with a 40% MMAT score did not yield any unique themes or codes, suggesting that all synthesis findings are supported by moderate to high-quality studies.

### Synthesis findings

Thematic synthesis identified seven clusters and an overarching phenomenon: starting the diagnostic workup for dementia is a delicate process. This phenomenon is influenced by:

(1) complexities arising from the nature of dementia
(2) interaction with the patient and family
(3) individual determinants of PCPs
(4) expectations of PCPs regarding the consequences of a diagnosis
(5) factors related to the healthcare system
(6) societal factors

**Figure 2:**
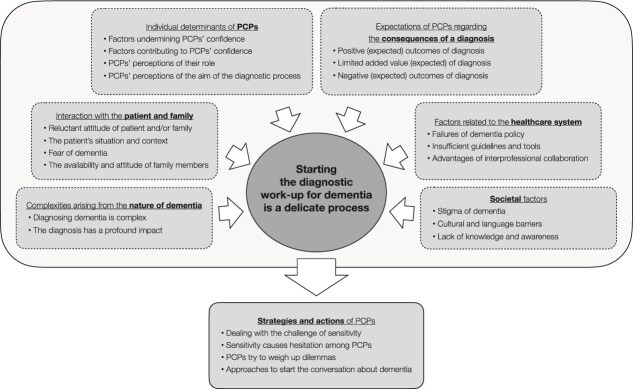
Primary care practitioners’ considerations and attitudes during the process from suspicion of cognitive decline to deciding to initiate diagnostic testing.

Together these factors form the strategies and actions of PCPs (7) whether or not to start the diagnostic workup for dementia (Figure 2 and Table 2). See Appendix 3 in the Supplementary Data for illustrative quotes.

*Cluster 1: Complexities arising from the nature of dementia.*

**Diagnosing dementia is complex.** Dementia and its diagnostic process are described by PCPs as nuanced, slow to evolve, dynamic, unpredictable and complex.

GP: ‘The act of diagnosis is really not just a case of gathering a few facts together, or even conducting a mini-mental test and giving a score out of thirty, and doing a range of blood tests and a scan and “there we have it, there’s the diagnosis”. That is the kind of biomedical understanding of how one would make the diagnosis, but in practice, dementia is a very complex problem which impacts on many people, all of whom have a stake in what is going on’. [15]

Family Physician (FP): ‘[Dementia,] it’s a moving target. It dynamically changes on a week-to-week basis’. [27]

**The diagnosis has a profound impact.** PCPs note that dementia has a profound impact. The diagnosis is considered significant, making it essential to get the diagnosis right.

GP: ‘It is a loaded diagnosis; it is sometimes better to have a broken leg, or better to have high blood pressures. And of course it doesn’t only affect the patient but their families or carers’. [35]

*Cluster 2: Interaction with the patient and family.*

**Table 3:** Characteristics of included studies

*First author (year), country*	*Study design* ^*a*^	*Study aim*	*Participants*	*Setting*	*MMAT* ^*b*^
Abe (2021), Japan and United States	Qualitative approach using semi-structured interviews in person, telephone or video	To explore the practices and perspectives of primary care physicians on the mutually common problem of diagnosing dementia in Japan and the United States	48 primary care physicians including family physicians and internists (United States: *n* = 24, Japan: *n* = 24)	Primary care setting	100%
Apesoa-Varano (2011), United States	Qualitative approach, secondary analysis of semi-structured interviews in person or telephone	To (i) explore how PCPs experience and approach the ongoing care of patients with Alzheimer’s disease and (ii) describe how this care unfolds from the physicians’ perspectives	Forty primary care physicians	Primary care setting	80%
Bature (2018), United Kingdom	Qualitative approach using semi-structured interviews	To explore the perspectives of GPs as to the factors that may be responsible for the late diagnosis of the disease as well as their recommendations to circumvent these	Seven general practitioners	Primary care setting	60%
Bryant (2021), Aboriginal and Torres Strait Island, Australia	Qualitative approach using semi-structured telephone interviews	To explore, from the perspective of care providers in the ACCHS sector, and across urban, regional and remote communities, current processes for dementia diagnosis and ongoing care, and barriers and enablers to high quality dementia care	One aboriginal health worker, two general practitioners, two registered nurses, one senior medical officer	Community/primary care setting, Aboriginal people	100%
Cahill (2008), Ireland	Mixed methods approach using a survey and focus group	To examine five key research questions, namely: (i) who is responsible for the late presentation of dementia in Ireland?; (ii) how long must GPs wait to access Geriatric, Old Age Psychiatry and Neuropsychological services?; (iii) what are the main barriers they experience attempting to diagnose dementia?; (iv) might financial incentives improve detection rates; and (v) what type of specific training and diagnostic guidelines might assist rural based GPs regarding dementia?	Focus group: seven general practitioners Survey: 300 general practitioners	Primary care setting	80%
Chithiramohan (2019), United Kingdom	Qualitative approach using semi-structured in depth interviews in person	To explore GPs’ views concerning barriers to diagnosing dementia six years after the introduction of QOF incentives, discuss assumptions underpinning these views and explore the impact of these barriers on clinical practice	Thirteen general practitioners	Primary care setting	100%
Constantinescu (2018), Canada	Qualitative approach using semi-structured focus groups	To explore rural family physicians’ experiences in caring for patients with dementia	Sixteen family physicians	Primary care setting	100%
Crombie (2024), Australia	Mixed methods approach, semi-structured interviews in person	To explore GP understanding of, and confidence and attitudes towards management of dementia in the rural context and (2) to design and pilot a dementia-specific GP training program in a single practice-group setting	Sixteen general practitioners	Primary care setting	100%
Dhedhi (2014), United Kingdom	Qualitative approach using narrative in depth interviews in person	To explore, from a narrative perspective, how the notion of ‘timeliness’ is constructed in practice, and how GPs account for the decisions they make about the diagnosis of dementia	Seven general practitioners	Academic department of primary care and public health	100%
Foley (2017), Ireland	Qualitative approach using semi-structured in-depth interviews in person	To explore GPs’ dementia care educational needs, by analysing information gathered from a variety of relevant sources, in order to inform the development of a primary care dementia educational program	Fourteen general practitioners	Primary care setting	100%
Gibson (2021), Australia	Qualitative approach using focus groups	1. Identify the PN roles in dementia care provision 2. Understand the barriers and enablers influencing the role of the PN in dementia care provision	Thirty-six primary care nurses (eight focus groups)	Primary care setting	80%
Gong (2023), China	Qualitative approach using focus groups and in-depth interviews in person	To explore the reasons that hindered the provision of dementia screening and management services by GPs in general practice	Focus groups: 30 general practitioners In-depth interviews: 22 general practitioners	Primary care setting	100%
Gove (2015), England	Qualitative approach using semi-structured in depth telephone interviews	To explore how GPs’ perceptions of dementia map onto current conceptualizations of stigma, how GPs understand the role of stigma in delaying timely diagnosis and to consider the implications of these findings for the involvement of GPs in attempts to tackle the stigma of dementia	Twenty-three general practitioners	Primary care setting	100%
Hansen (2008), Australia	Qualitative approach using semi-structured interviews in person and focus groups	To explore general issues related to dementia care in general practice	Twenty-four general practitioners	Primary care setting	80%
Le Huynh-Truong (2023), Vietnam	Qualitative approach semi-structured interviews in person and focus groups	To understand the variables that inform the practice of dementia care for community HCPs in Vietnam	Twenty-three physicians, physician’s assistants and community nurses	Community setting	80%
Iliffe (2005), United Kingdom	Qualitative approach using group work discussions	To identify barriers to the recognition of and response to dementia in primary care as perceived by general practitioners and highlights areas of information and training need	One hundred and forty four general practitioners	Primary care setting	40%
Kaduszkiewicz (2008), Germany	Mixed methods approach using semi-structured in-depth interviews	To investigate differences between GPs and specialists (neurologists and psychiatrists) in the German ambulatory care system concerning the disclosure of the diagnosis of dementia	30 general practitioners	Primary care setting	60%
Lahjibi-Paulet (2012), France	Qualitative approach using semi directed interviews in person	To analyse the attitudes and perceptions of GPs in France, most particularly in Paris, through a discussion of their clinical practices regarding the diagnosis and management of ad	Twenty-five general practitioners, of which 24 interviewed and 20 analysed interviews	Primary care setting	60%
Leung (2020), China	Mixed methods approach using focus groups	To assess primary care physicians’ knowledge and attitudes about the early detection of dementia in Hong Kong	Thirty-one primary care physicians	Primary care setting	80%
Lindeberg (2022), Sweden	Qualitative approach using semi-structured interviews	To investigate Swedish clinical professionals’ experiences of diagnostic pathways in dementia, focusing on the assessment of cognitive and communicative abilities	Four general practitioners	Primary care setting	80%
Linden (2024), The Netherlands	Qualitative approach using semi-structured telephone interviews	To provide greater insight into the current decision-making process on diagnostic testing for dementia by exploring the expectations, needs and experiences of patients with memory complaints, significant others and general practitioners	Fourteen general practitioners, two practice nurses	Primary care setting	100%
Moore (2013), Ireland and Sweden	Qualitative approach using semi-structured in depth interviews in person	To explore the attitudes of Irish and Swedish GPs to the diagnosis and disclosure of dementia to patients; to investigate GP under-graduate/post-graduate training in dementia; to examine the post-diagnostic support services available to GPs in both countries and to investigate the extent to which dementia is perceived as stigmatizing	Nine general practitioners	Primary care setting	60%
Murphy (2014), Australia	Qualitative approach using semi-structured interviews in person or telephone	To explore GPs’ reported practice in diagnosing and managing dementia and to describe, in theoretical terms, the proposed explanations for practice that was and was not consistent with evidence-based guidelines	Thirty general practitioners	Primary care setting	100%
Palumbo (2020), New Zealand and United States	Qualitative approach using semi-structured interviews in person	To explore and describe the use of national dementia care guidelines by primary care providers in a selected region of New Zealand	Five general practitioners, six nurse practitioners	Primary care setting	80%
Phillips (2012), Australia	Qualitative approach using semi-structured interviews in person	To explore Australian GPs’ perceptions of disclosing the diagnosis of dementia	Forty-five general practitioners	Primary care setting	100%
Prins (2016), The Netherlands	Qualitative approach using semi-structured interviews in person	To explore Dutch GPs’ perceptions of their current position in diagnosing dementia, their reasons for referral to secondary care, and views on the future diagnostic role of GPs	Eighteen general practitioners	Primary care setting	60%
Robinson (2008), Australia	Qualitative approach using focus groups	To reveal views about dementia diagnosis derived from a larger study of information needs of carers of people with dementia in Tasmania, Australia	Seven general practitioners	Primary care setting	80%
Sagbakken (2018), Norway	Qualitative approach using in depth interviews in person and focus groups	To explore challenges involved in identifying, assessing and diagnosing people with cognitive impairment/dementia who have different linguistic and cultural backgrounds	Two general practitioners	Primary care setting, Immigrants	100%
Sideman (2023), United States	Qualitative approach using interviews by video	To describe PCP perspectives on their role in dementia diagnosis and care	Thirty medical doctors, six nurse practitioners, three doctors of osteopathic medicine	Primary care setting	100%
Steiner (2020), Australia	Qualitative approach using semi-structured in depth interviews by telephone	To ensure the region-specific needs of the memory clinic were considered by co-creating the model of care with local GPs, community health care workers, local government, and local community members including seniors, carers and people with dementia	Twenty general practitioners	Primary care setting	100%
Tromp (2021), The Netherlands	Qualitative approach using semi-structured interviews in person	To explore the ethical considerations that shape current clinical practice regarding early ad diagnostics and the use of biomarkers	Five general practitioners	Primary care setting	100%
Vissenberg (2018), The Netherlands	Mixed methods approach using focus groups	To describe the barriers for providing primary care to immigrant patients (Turkish, Moroccan and Surinamese) with dementia from the perspectives of GPs	Fourteen primary care physicians (three focus groups)	Primary care setting, Immigrants	80%
Wangler (2020), Germany	Qualitative approach using semi-structured interviews	To determine the predictors for the quality and effectiveness of general practitioner dementia care as holistically as possible	Forty-one general practitioners	Primary care setting	40%

Abbreviations: PCP = primary care practitioner, GP = general practitioner, PN = practice nurse, ad = Alzheimer’s disease

^a^In case of a mixed-methods design, details are provided on the qualitative design only.

^b^MMAT: percentage of qualitative quality criteria met.

**Reluctant attitude of patient and/or family.** PCPs often face challenges when dealing with a reluctant attitude of the patient and/or family. Patients may deny, hide or normalise symptoms, and the disease symptoms can cause limited insight. Individuals may avoid seeking help, or are reluctant to accept a diagnosis. Related to this, PCPs find it difficult to get a patient to agree to be tested.

GP: ‘Well, one man denies it completely...and a partner who also really covers it up. So they do not want it’. [48]

**The situation of the patients and their context**. This also affects the PCPs’ actions. PCPs value the patient’s need, wish and right (not) to know. Additionally, patient characteristics and symptom severity influence how urgent PCPs feel a diagnosis is. The patient’s context, such as living in a rural versus urban area, educational background and awareness of dementia, overall health and social circumstance, also play a role in the PCPs’ actions.

GP: ‘Rural patients typically don’t want to have to go into a city, they don’t want to have to live in an apartment [...]. It’s a tougher diagnosis to give an 85-year-old farmer than an 85-year-old executive guy [...]. You don’t just take the guy away from his farm’. [27]

**Fear of dementia.** PCPs describe that patients and family members often experience fear regarding the diagnosis, worries about losing independence or concerns about worst-case scenarios.

GP: ‘She said that they feared becoming more and more idiotic’. [36]

Additionally, PCPs report that receiving a diagnosis is emotionally difficult for the patient and family.

**The availability and attitude of family members.** The involvement of family members is crucial. They are usually the ones that report symptoms. Also, the history provided by family is essential information for eventually making the diagnosis, and the patient’s social support system is an important factor in the decision-making process.

*Cluster 3: Individual determinants of PCPs.*

Factors related to the PCP that may influence the initiation of the diagnostic workup include their confidence, perceptions of their role and their perceptions of the aim of the process.

**Factors undermining or contributing to PCP confidence**. Lack of knowledge, training or education undermines the PCP’s confidence. PCPs quote not feeling comfortable making the diagnosis of dementia.

*GP: ‘We’re certainly not in our comfort zone with this’.* [29].

Additionally, PCPs may feel a lack of confidence due to the complexity of interpreting cognitive test results. They find it challenging to differentiate between dementia and depression, or normal ageing and cognitive impairment. Additionally, conflicting information from different sources can further undermine their confidence.

GP: ‘Sometimes it’s dubious, when a person can seem quite confused and still perform quite well on these tests. So, it’s not always totally clear, or easy, to make a diagnosis. When it hasn’t progressed that far. [...] There’s often denial and some verbal discussions when you see a person with dementia and a relative together. Where they have totally different views on what issues there are’. [32]

Conversely, PCPs feel more comfortable in obvious cases of dementia and with back-up access to specialists.

**PCPs’ perceptions of their role**. Many studies highlight that PCPs believe a specialist should make or confirm the diagnosis. Nevertheless, some PCPs see themselves as uniquely positioned to guide patients through the diagnostic trajectory. PCPs use metaphors like ‘fellow traveller’ or ‘we take this together’ [15].

**PCPs’ perception of the aim of the process.** PCPs have different views regarding the aim of the diagnostic process. However, a common aim is to prioritise the patient’s well-being or safety over confirming a diagnosis.

GP: ‘I look after you, you are my concern and less of a concern is which label I use for what you have’ [15]

PCPs also strive to meet the needs and preferences of patients and their family, and they try to encourage and support patients in living independently and ensuring their safety at home.

*Cluster 4: Expectations of PCPs regarding the consequences of a diagnosis.*

PCPs have a wide range of expectations regarding what a diagnosis will provide. Positive consequences include increased access to care and support, future planning and the possibility for patients to decide for themselves. PCPs also suggest that naming can lead to clarification and de-mystification. Other benefits noted by PCPs include counselling or guidance, appropriate medication and improvements in well-being, quality of life and safety.

GP: ‘It gives a name to what’s happening to them if they’ve noticed symptoms and a justification for any deficits they’re noticing. It also gives them time. If it’s mild cognitive impairment they’ve got time to put in strategies and to make some plans for the future in an informed way, which they can’t if it isn’t addressed’. [41]

However, PCPs also mention the limited added value of a diagnosis. Many point out that there is no clear effect of treatment, that the availability of care resources can be limited, and that specifying the type of dementia may not add value. In some cases, family and neighbours already provide adequate care. Some PCPs hesitate to diagnose dementia because the patient may not understand the diagnosis and its implications. PCPs report negative outcomes associated with diagnosing dementia, such as the emotional distress for the patient and family and potential legal or financial issues. They express concerns about not wanting to medicalise the ageing process. Another challenge addressed by PCPs is the need to revoke a patient’s driving licence in certain cases.

FP: ‘You know that now the driver’s license is going to have to be taken away and you know that a lot of these people have to move from where they’re living somewhere else at some point, families get torn apart’. [27]

*Cluster 5: Factors related to the healthcare system.*

PCPs report several factors related to the healthcare system that could hinder or facilitate the initiation of the diagnostic workup for dementia.

**Failures of dementia policy.** Hindering factors include time constraints in general practice, lack of care available, lack of funding and dementia being a low-priority health condition.

**Insufficient guidelines and tools.** PCPs experience that guidelines or referral mechanisms are absent or not clear. Additionally, diagnostic tools are time-consuming and provide insufficient guidance. PCPs express a need for the availability of an accepted, valid and practical tool to diagnose dementia.

GP: ‘[referral] vague concept. The pathway is unclear as to where exactly to go and what to do next for the positive patients [who screen out]’. [30]

**Advantages of interprofessional collaboration.** Facilitating factors are a strong team culture and effective interprofessional collaboration, particularly with practice nurses. PCPs indicate these are pivotal in initiating the diagnostic trajectory. Collaborating closely with a practice nurse experienced in dementia care offers several advantages, such as extra time, the ability to visit a person in their home environment for gathering additional information and fostering a nurse–patient relationship that is often perceived as less intimidating than the physician–patient dynamic.

GP: ‘Together we can offer a much better diagnostic work-up at home and see what goes wrong in the home situation, than at a specialised memory clinic. At home you observe so much more and this is so valuable. And as a GP or practice-based nurse specialist, you have much easier access and for the patient it’s less threatening. The patient and their family often give back that they find this a very welcome way for a diagnostic work-up’. [50]

*Cluster 6: Societal factors.*

**Stigma of dementia**. The sensitivity of starting diagnostic testing is partly caused by the stigma associated with the condition. PCPs observe that communities avoid using the terms ‘dementia’ or ‘Alzheimer’s’.

**Cultural and language barriers.** Cultural perspectives on dementia can vary, which affects how sensitive a dementia diagnosis is. In some cultures, dementia may be viewed as taboo or linked to bad karma. Additionally, cultural and language barriers, along with low literacy levels, can complicate accurate cognitive assessments. Existing cognitive tests may not be suitable for all populations.

GP: ‘Literacy is another problem especially with the older generation. So, I find it difficult to know what tools to use to diagnose a dementia […] you use your MMSE and it will ask you like when was World War One and they just won’t know, not because …it’s because they never knew in the first place. So I don’t think it is tailored to different languages and cultures’. [35]

**Lack of knowledge and awareness.** PCPs also highlight that limited knowledge and awareness in society can lead to a lack of understanding regarding the condition and its symptoms. Some PCPs indicate there is a widespread belief that memory problems are a normal part of ageing which limits help-seeking.

*Cluster 7: Strategies and actions of PCPs.*

We identified the strategies and actions that PCPs use to navigate the delicate process of starting the diagnostic workup for dementia. These strategies vary from proactively addressing the challenge of sensitivity to adopting a more hesitant, reactive approach. PCPs describe ways to initiate the conversation about this sensitive topic, as well as the dilemmas they face while weighing the options.

**Dealing with the challenge of sensitivity.** To deal with the sensitivity of discussing diagnostic testing, PCPs take it slowly, gradually introduce the topic, search for the ‘right’ moment, emphasise their role in helping the person rather than focusing on the label, and communicate that the patient is in charge during the process.

GP: ‘Usually I tell them “It is not that we will decide everything for you, you always remain in charge”’ [9]

PCPs find that building trust or assessments in the home environment helps to manage sensitivity. Some PCPs avoid the burden of delivering bad news by referring to a specialist at the hospital or memory clinic. Additionally, PCPs may avoid using terms like ‘dementia’ or Alzheimer’s’.

**Approaches to start the conversation about dementia.** The analysis identified several ways in which PCPs can initiate a conversation about dementia, even in the absence of a direct request for help from the patient. PCPs find it useful to have an opening to address concerns about dementia. For instance, PCPs use changes observed during consultations as a starting point for the conversation. A proactive approach, such as conducting routine health checks or screening, prompts discussions about dementia. Furthermore, continuity of care and a holistic approach encourages looking beyond known diseases, and enable recognition of and conversations about dementia.

PCP: ‘We tend to know the people [with suspected dementia] that we need to tap on the shoulder to bring in [...] and talk to’. [27]

**Sensitivity causes hesitation among PCPs.** In situations where PCPs are more hesitant to address this delicate topic, they may be more cautious to label the patient. They may find it difficult how and when to bring up the topic, and fear that it might harm the physician–patient relationship. PCPs report that cognitive testing is often distressing, embarrassing or uncomfortable. The hesitancy leads to a reactive approach to diagnosis. For example, PCPs tend to rely on patients to report symptoms or adopt a ‘wait-and-see’ approach until problems become evident.

GP: ‘I avoid conflict with the patient. I don’t want him to get angry and I don’t want to lose him as my patient. It’s not for financial reasons; every patient I lose will be replaced by another, but for me mutual trust is very important. And I think that such a disclosure can be very offending for the patient’ [36]

**PCPs try to weigh up dilemmas.** In this complex decision-making process, PCPs try to weigh various dilemmas, including the benefits and potential negative consequences of a diagnosis. Their evaluation depends partly on their own expectations regarding the consequences of a diagnosis (cluster 6). Additionally, PCPs value shared decision-making and aim to align their actions with the patient’s and family’s preferences. However, this may require negotiating conflicting priorities and expectations. Furthermore, PCPs sometimes find it challenging to balance patient autonomy with the need to prevent risks or crises.

GP: ‘I think it is a negotiation as to what one can do. So, you can always negotiate harder and I certainly could have negotiated harder [in this particular case] but I would prefer—I mean, maybe it’s a personal style—I certainly would prefer that, you know, they come, or eventually come round to your view. Now, the catch with that is that sometimes what happens is you get a crisis. You could say ‘Well, you could have intervened earlier’. Yeah! But that then would have been counter to providing him with any particular form of, you know, autonomy. So, that’s a constant struggle, just knowing, ‘could I have done that?’ ...it’s a constant struggle. I mean, it’s difficult to know, because how would I know anyway whether it was a better or worse decision?’ [15]

## Discussion

This is the first comprehensive review of the considerations and attitudes of HCPs on starting the diagnostic workup for dementia. All included studies were conducted in the primary care or community setting. The main phenomenon observed is that starting the diagnostic workup for dementia involves a delicate process. This process is influenced by various factors, including: *‘*complexities arising from the nature of dementia’ (profound impact of dementia, complexity of the diagnostic process), *‘*interaction with the patient and family*’* (e.g. their fear of dementia, reluctant attitude, individual situation), ‘individual determinants of PCPs’ (e.g. their perceptions of their role and the aim of the process, their confidence), *‘*expectations of PCPs regarding the consequences of a diagnosis, ‘factors related to the healthcare system’ (e.g. time constraints, lack of funding, lack of clear guidelines or referral mechanisms, diagnostic tools providing insufficient guidance) and ‘societal factors’ (stigma, cultural and language barriers, knowledge and awareness). To navigate this sensitive process of starting the diagnostic workup for dementia, PCPs employ various strategies. These range from proactively addressing the sensitivity to adopting a more hesitant, reactive approach. Some PCPs incorporate routine health checks or look for openings to broach the topic of dementia during consultations. They try to weigh the dilemmas they encounter after becoming aware of dementia signs.

### Interpretation of findings—what could be improved

This review highlights key factors to target for improving the timing of dementia diagnosis. Our analysis shows that PCPs’ confidence in diagnosing dementia is a recurring barrier, consistent with previous research [57, 58]. Beyond calls for increased knowledge and training to enhance confidence, our findings demonstrate that PCPs struggle with contradictions between information sources, and conflicting priorities and expectations. Previous research indicates that patients and their families also face conflicting interpretations of symptoms [59]. Diagnostic decision aids may help PCPs, individuals with cognitive symptoms and their families to navigate this complexity [60]. Additionally, confidence-related areas to address include cognitive test interpretation, distinguishing between dementia, depression and normal ageing, and enhancing PCPs’ awareness of the ethical dilemmas involved in diagnosing dementia, and how to address these. Future research should examine how to provide more guidance and support for PCPs in managing these challenges.

Regarding the PCPs’ roles in diagnosing dementia, healthcare system stakeholders, such as health insurers, guideline developers, educators and policymakers, must ensure consistent communication and clarity. This would encourage PCPs to diagnose more common presentations of dementia and know when to refer to a specialist. This understanding is also important for society, as, for example, in the Netherlands, some patients believe GPs are unqualified to be involved in the diagnostic process, let alone diagnose dementia [59]. Other societal issues that need to be addressed include stigma, fear of a dementia diagnosis (along with the associated worst-case scenarios) and lack of knowledge and awareness. Responsible parties (e.g. governmental organizations or national Alzheimer’s associations) should communicate what a diagnosis can offer, and that GPs are qualified to diagnose dementia. Instead of isolating individuals with dementia, we should engage with them as fellow human beings and encourage their participation in society. This aligns with research efforts to change societal perceptions [61, 62].

Our findings highlight the importance of teamwork and collaboration in balancing the needs of patients and their families. Insurance companies and policymakers should provide sufficient time and funding to proactively manage this process and collaborate with practice nurses or physicians specialised in caring for older adults outside the hospital setting. For example, in the Dutch healthcare system, some GPs collaborate with physicians who traditionally work in nursing homes [63]. They can dedicate extra time with patients, conduct home visits, adopt a less intimidating role or serve as independent experts. For patients with a typical presentation of dementia, this collaboration can lessen the physical, emotional and financial burden on them, their family and the healthcare system by decreasing reliance on memory clinics. Moreover, working with practice nurses may allow for proactive detection and monitoring of cognitive problems in frail individuals. GPs perceive such a proactive approach as helpful for managing cases where patients may not seek care, for anticipating care needs and preventing crises [11]. Similarly, our findings align with previous research indicating that routine health checks facilitate timely dementia diagnosis by making it easier to discuss the sensitive topic [64, 65]. Furthermore, this is supported by research from the perspective of society, where community-dwelling adults aged 50 and older expressed a greater willingness to seek help if dementia checks were included in their GP’s routine examinations [66].

### Interpretation of findings—what must be accepted

Our findings also address more challenging areas for improving the timeliness of dementia diagnosis. We found that the complexity of the diagnostic process, along with individual preferences and the situation of the patient, family and PCPs largely influence starting diagnostic testing. Consequently, there is no one-size-fits-all approach that can be encompassed within guidelines. This likely explains the poor adherence to guidelines observed among PCPs in previous studies [67, 68]. Therefore, it is essential for guidelines and training to communicate the dynamics, difficult considerations and decisions that need to be addressed, as well as the importance of discussing these matters explicitly with patients and their families.

In addition, the complexity and the profound impact of a diagnosis as shown by the results explain the uncertainty that clinicians may experience. This raises the question of whether we should accept to a certain degree that PCPs refer low-complexity patients to specialists [67, 69]. If collaboration with a practice nurse or a physician specialised in caring for older adults is not feasible, we may need to acknowledge that patients are sometimes referred to memory clinics where the diagnosis is already clear and does not require further investigation. Moreover, these low-complexity referrals can serve as learning experiences, for example through teleconsultations or other formats that encourage clinical reasoning [70, 71]. This approach might eventually reduce the number of unnecessary referrals. Similarly, in cases where patients fear the diagnosis, deny their cognitive decline or prefer not to know their diagnosis, it may be necessary to accept that a crisis must occur before action can be taken. However, the interactions of PCPs with patients and their families might change if more effective treatments for dementia become available [72].

### Lack of research on hospital-based HCPs without expertise in cognition

Notably, our search did not identify any studies addressing the considerations of hospital-based HCPs. Consequently, the results are not transferrable to hospital settings. It is important for HCPs, not just those in primary care, to consider cognitive functioning, as cognitive dysfunction impacts care needs and decision-making [10, 73, 74]. This highlights the need for future research focusing on perspectives of hospital-based HCPs. HCPs in hospital settings typically have shorter interactions with patients. This could present challenges for recognising and discussing dementia due to limited insight into the patient’s circumstances. Conversely, less personal interaction might facilitate discussions about sensitive topics, as there could be less concern about breaching a long-established trust [11].

### Strengths and limitations

This review provides a comprehensive understanding of the considerations of PCPs on starting the diagnostic trajectory for dementia through qualitative synthesis. It builds on previous research reviewing quantitative studies related to this topic [57]. Our findings not only confirm the barriers to optimal dementia care and show their persistence in recent studies, they also provide a deep understanding of the barriers and facilitators [57]. Another strength is the gained insight into how some PCPs address these challenges. The credibility of the analysis may be affected by only coding the first 15% of articles up to the preliminary coding tree independently. However, the strengths of our analysis process include the iterative discussions and refinement of codes and themes (reflexivity), and the multidisciplinary nature of the research team (investigator triangulation). Most included studies had methodological quality rated as moderate to high. The lower quality studies did not negatively affect our data synthesis since they did not introduce new themes or codes. However, the results of this review may not be universally applicable to all healthcare settings because the data originate from diverse countries, cultures and healthcare systems. Our findings demonstrate differences, for example, in one culture or country, there may not even be a word for dementia, while in others, it is a standard part of routine health assessments.

## Conclusion

PCPs perceive that starting the diagnostic workup for dementia is a delicate process. A wide variety of factors influences this process and complicates a deliberate and shared decision with patients and their families. Some complexities include the fear of dementia, the stigma attached to it and the reluctant attitude of patients or their families. The different strategies that PCPs use to address the complexities cannot be captured by a single right approach. Recommendations include ensuring consistent communication and clarity regarding the roles of PCPs, encouraging interprofessional collaboration with practice nurses, enhancing confidence in managing conflicting priorities and interpreting cognitive test results.

## Supplementary Material

Supplementary_materials_afaf176

## Data Availability

Data sharing is not applicable to this article as no new data were created or analysed in this study.
