# Supplementary material for: From suspicion of cognitive decline to dementia diagnosis: a systematic review of healthcare professionals’ considerations and attitudes
Source: Age Ageing. 2025 Jun 24;54(6):afaf176. doi: 10.1093/ageing/afaf176 (PMC12206097; doi:10.1093/ageing/afaf176)
Supplement: Supplementary_materials_afaf176 [file supplementary_materials_afaf176.docx]

**From suspicion of cognitive decline to dementia diagnosis: a systematic review of healthcare professionals’ considerations and attitudes**

**SUPPLEMENTARY DATA**

Appendix 1. Search Strategy PubMed *page 2*

Appendix 2. Results of the quality assessment

using the Mixed Methods Appraisal Tool *page 3*

Appendix 3. Themes derived from data synthesis *page 6*

with illustrative quotes.

Appendix 1. Search Strategy PubMed

| **Concepts** | **Search terms** |
| --- | --- |
| #1 Dementia | ("Dementia"[Mesh] OR dementi*[tiab] OR Alzheimer*[tiab]) |
| #2 Healthcare professionals | ("Anesthetists"[Mesh] OR anesthetist*[tiab] OR anesthesiologist*[tiab] OR anaesthesist*[tiab] OR anaesthetist*[tiab] OR anesthesist*[tiab] OR anaesthesiologist*[tiab] OR "Medical Staff"[Mesh] OR “medical staff*”[tiab] OR “medical specialist*”[tiab] OR resident*[tiab] OR  “medical registrar*”[tiab] OR “hospital registrar*”[tiab] OR "Nurses"[Mesh] OR "Nursing Staff"[Mesh] OR nurs*[tiab] OR "Physicians"[Mesh] OR physician*[tiab] OR doctor*[tiab] OR cardiologist*[tiab] OR dermatologist*[tiab] OR endocrinologist*[tiab] OR gastroenterologist*[tiab] OR hepatologist*[tiab] OR “general practitioner*”[tiab] OR gynecologist*[tiab] OR nephrologist*[tiab] OR oncologist*[tiab] OR ophthalmologist*[tiab] OR otolaryngologist*[tiab] OR otologist*[tiab] OR physiatrist*[tiab] OR “primary care provider*”[tiab] OR pulmonologist*[tiab] OR rheumatologist*[tiab] OR surgeon*[tiab] OR orthopedists*[tiab] OR urologist*[tiab] OR gps[tiab] OR “gp’s”[tiab] OR "healthcare provider*"[tiab] OR “healthcare professional*”[tiab] OR “health care professional*”[tiab] OR "health care provider*"[tiab] OR practitioner*[tiab]) |
| #3 Considerations and attitudes | ("Attitude"[Mesh:NoExp] OR attitude*[tiab] OR "Attitude of Health Personnel"[Mesh] OR sentiment*[tiab] OR opinion*[tiab] OR "Judgment"[Mesh] OR judgment*[tiab] OR judgement*[tiab] OR "Decision Making"[Mesh] OR “decision making*"[tiab] OR "Clinical Decision-Making"[Mesh] OR  decisionmaking*[tiab] OR “choice making*”[tiab] OR “choice behaviour*”[tiab] OR “choice behavior*”[tiab] OR perception*[tiab] OR experience*[tiab] OR understanding*[tiab] OR view*[tiab] OR perspective*[tiab] OR preference*[tiab] OR “decision process*”[tiab] OR reason*[tiab] OR argument*[tiab] OR belief*[tiab] OR practice*[tiab] OR standpoint*[tiab] OR consideration*[tiab] OR motivation*[tiab] OR motive*[tiab] OR thought*[tiab]) |
| #4 Diagnosis | ("Diagnosis"[Mesh:NoExp] OR "Delayed Diagnosis"[Mesh] OR "Early Diagnosis"[Mesh:NoExp] OR diagnos*[tiab] OR ((identif*[tiab] OR recogn*[tiab] OR detect*[tiab]) AND (cognit*[tiab] OR memory[tiab] OR dementi*[tiab] OR Alzheimer*[tiab]))) |
| Combination of search strings | #1 AND #2 AND #3 AND #4 |
| Limiters | Published from 2005-2024 |

Appendix 2. Results of the quality assessment using the Mixed Methods Appraisal Tool

| **First author** | 1.1 | 1.2 | 1.3 | 1.4 | 1.5 | 4.1 | 4.2 | 4.3 | 4.4 | 4.5 | 5.1 | 5.2 | 5.3 | 5.4 | 5.5 | Total % (qualitative part) | Explanation |
| --- | --- | --- | --- | --- | --- | --- | --- | --- | --- | --- | --- | --- | --- | --- | --- | --- | --- |
| Abe | Y | Y | Y | Y | Y |  |  |  |  |  |  |  |  |  |  | 100% |  |
| Apesoa-Varano | Y | ? | Y | Y | Y |  |  |  |  |  |  |  |  |  |  | 80% | 1.2 Missing details on interview guide development, interviewer experience, and sampling strategy. |
| Bature | Y | N | ? | Y | Y |  |  |  |  |  |  |  |  |  |  | 60% | 1.2 No explanation of the purposive sampling strategy's purpose, short interview duration without justification, no details on who conducted interviews or researchers' experience; 1.3 No details on who conducted the analysis or researchers' qualitative research experience, no explanation of the framework used or how it was chosen. |
| Bryant | Y | Y | Y | Y | Y |  |  |  |  |  |  |  |  |  |  | 100% |  |
| Cahill | Y | Y | ? | Y | Y | Y | Y | Y | Y | ? | Y | Y | Y | Y | Y | 80% | 1.3 Lacks information on the coding process (development of codes, categories, themes, roles of facilitators); 4.2 Possibility of a biased sample, so representativeness unclear. |
| Chithiramohan | Y | Y | Y | Y | Y |  |  |  |  |  |  |  |  |  |  | 100% |  |
| Constantinescu | Y | Y | Y | Y | Y |  |  |  |  |  |  |  |  |  |  | 100% |  |
| Crombie | Y | Y | Y | Y | Y | Y | ? | Y | ? | Y | Y | Y | Y | Y | Y | 100% | 4.2 Likely biased due to few respondents, aim was not representativeness; 4.4 Not described. |
| Dhedhi | Y | Y | Y | Y | Y |  |  |  |  |  |  |  |  |  |  | 100% |  |
| Foley | Y | Y | Y | Y | Y |  |  |  |  |  |  |  |  |  |  | 100% |  |
| Gibson | Y | ? | Y | Y | Y |  |  |  |  |  |  |  |  |  |  | 80% | 1.2 Participants were from a dementia education training, leading to a selection with a specific interest or experience in dementia, making it difficult to identify the general role of the PN (which was the research question). |
| Gong | Y | Y | Y | Y | Y |  |  |  |  |  |  |  |  |  |  | 100% |  |
| Gove | Y | Y | Y | Y | Y |  |  |  |  |  |  |  |  |  |  | 100% |  |
| Hansen | Y | ? | Y | Y | Y |  |  |  |  |  |  |  |  |  |  | 80% | 1.2 Little information on participant variation, interviewers’ backgrounds and qualitative experience unknown, second focus group chosen due to recruitment issues; 1.3 Coding done by one author, only the discussion on themes with multiple researchers. |
| Le Huynh‐Truong | Y | ? | Y | Y | Y |  |  |  |  |  |  |  |  |  |  | 80% | 1.2 Unknown who the interviewers were, unclear backgrounds and qualitative research experience, limited explanation of sampling strategy. |
| Iliffe | Y | ? | ? | N | Y |  |  |  |  |  |  |  |  |  |  | 40% | 1.2 No mention of discussions being recorded and transcribed, unknown background of moderator, unclear how it was determined that sufficient data had been collected; 1.3 No clear analysis method, limited description of analysis; 1.4 Interpretation of results not supported by quotes. |
| Kaduszkiewicz | Y | ? | Y | Y | N | Y | ? | Y | Y | ? | Y | Y | Y | Y | N | 60% | 1.2 Sampling strategy poorly explained, number of interviews not substantiated, no information about qualitative research experience of interviewers, and no information on development of the interview guide; 1.5 No specialists interviewed despite research question on differences, no explanation given; 4.5 Logistic regression analysis not explained in the methods section. |
| Lahjibi-Paulet | Y | N | N | Y | Y |  |  |  |  |  |  |  |  |  |  | 60% | 1.2 Interviewed GPs were a specific selection of medical educators, not appropriate to the broad research question, unknown who conducted the interviews and their experience, no justification for the sample size of 25, unclear development of the interview guide; 1.3 Coding and analysis done by a single person. |
| Lueng | Y | Y | ? | Y | Y | Y | Y | Y | Y | Y | Y | Y | Y | Y | Y | 80% | 1.3 No reference to scientific literature or description of thematic analysis steps. |
| Lindeberg | Y | ? | Y | Y | Y |  |  |  |  |  |  |  |  |  |  | 80% | 1.2 Interviewer’s background/experience not described, no explanation for development of interview guide, sample size, or cessation of data collection. |
| Linden | Y | Y | Y | Y | Y |  |  |  |  |  |  |  |  |  |  | 100% |  |
| Moore | Y | ? | N | Y | Y |  |  |  |  |  |  |  |  |  |  | 60% | 1.2 Unknown who conducted interviews and their background/experience, lack of justification for sample, snowball sampling used despite broad research question; 1.3 Single coder with limited cross-checking, not discussed with multiple researchers. |
| Murphy | Y | Y | Y | Y | Y |  |  |  |  |  |  |  |  |  |  | 100% |  |
| Palumbo | Y | N | Y | Y | Y |  |  |  |  |  |  |  |  |  |  | 80% | 1.2 Unknown who conducted interviews and their background/experience, purposive sampling with no description of what the goals were, and a change to snowball sampling without explaining why. |
| Phillips | Y | Y | Y | Y | Y |  |  |  |  |  |  |  |  |  |  | 100% |  |
| Prins | Y | ? | ? | Y | Y |  |  |  |  |  |  |  |  |  |  | 60% | 1.2 Purposive sampling with no description of what the goals were, study population mainly female GPs from one city; 1.3 Unknown who conducted coding, how many were involved, background not described. |
| Robinson | Y | Y | Y | N | Y |  |  |  |  |  |  |  |  |  |  | 80% | 1.4 Participants’ paraphrased statements displayed with key words in inverted commas, no quotes presented. |
| Sagbakken | Y | Y | Y | Y | Y |  |  |  |  |  |  |  |  |  |  | 100% |  |
| Sideman | Y | Y | Y | Y | Y |  |  |  |  |  |  |  |  |  |  | 100% |  |
| Steiner | Y | Y | Y | Y | Y |  |  |  |  |  |  |  |  |  |  | 100% |  |
| Tromp | Y | Y | Y | Y | Y |  |  |  |  |  |  |  |  |  |  | 100% |  |
| Vissenberg | Y | ? | Y | Y | Y | Y | Y | Y | ? | Y | Y | Y | Y | N | Y | 80% | 1.2 Unknown if data collection covers research question, due tot snowball sampling and not intentionally recruited until data saturation was reached, participant characteristics in focus groups not described; 4.4 Response rate 16%, no information on non-response participants. |
| Wangler | N | Y | ? | Y | N |  |  |  |  |  |  |  |  |  |  | 40% | 1.1 Research goal 'to determine the predictors for the quality and effectiveness' should be measured quantitatively; 1.3 Unknown who conducted coding, how many coders, their background, no explanation of the process or steps; 1.5 Results and interpretation focus mainly on the reserved or critical standpoint of GPs on dementia diagnostics, not linked to the research goal of determining predictors for quality and effectiveness of dementia care. |

N = no

Y = yes

? = can’t tell

Appendix 3. Themes derived from data synthesis with illustrative quotes.

| *Clusters, themes* | | *Quotes illustrative to the codes* |
| --- | --- | --- |
| CLUSTER 1: COMPLEXITIES ARISING FROM THE NATURE OF DEMENTIA | | |
| Diagnosing dementia is complex | | **Dementia presents in a dynamic and unpredictable course**  FP: “[Dementia,] it’s a moving target. It dynamically changes on a week-to-week basis and sometimes it’s hard to make the connection or the diagnosis.” (Constantinescu et al., 2018)  **The process is complex**  GP: “Cancer is almost cut and dried. You can do some tests and out comes cancer. Whereas dementia, it's like a “to and fro game” with the patient.” (Crombie et al., 2024)  GP: “The act of diagnosis is really not just a case of gathering a few facts together, or even conducting a mini-mental test and giving a score out of thirty, and doing a range of blood tests and a scan and ‘there we have it, there’s the diagnosis’. That is the kind of biomedical understanding of how one would make the diagnosis, but in practice, dementia is a very complex problem which impacts on many people, all of whom have a stake in what is going on.” (Dhedhi et al., 2014) |
| The diagnosis has a profound impact | | **Dementia is a significant diagnosis**  GP: “It is a loaded diagnosis; it is sometimes better to have a broken leg, or better to have high blood pressures. And of course it doesn’t only affect the patient but their families or carers . . .” (Chithiramohan et al., 2019)  GP: “such a significant diagnosis” (Phillips et al., 2012)  **Essential to get the diagnosis right**  GP: “you want to make sure … your facts are right” (Phillips et al., 2012) |
| CLUSTER 2: INTERACTION WITH THE PATIENT AND FAMILY | | |
| Reluctant attitude of patient and/or family | | **Challenging to get a patient agree to be tested**  GP: “But the real challenge here is to get people to come around eventually to having an assessment.” (Dhedhi et al., 2014)  **Denial by patient – hide or normalize symptoms, limited insight**  GP: “I think sometimes patients themselves try and normalize the concerns. I have patients who have frequently come to me and say well everyone gets forgetful a bit as they get older and also relatives.” (Bature et al., 2018)  GP: ‘‘People with dementia are very good, in the main, at hiding the problems’’ (Hansen et al., 2008)  **People do not seek help**  PCP: “I guess we never get self-identification. There might oc- casionally have people come in going my elderly relative isn't functioning very well and I want you to check him out. That doesn't seem to happen. It's more picked up incidentally.” (Bryant et al., 2021)  **Denial by patient – hide or normalize symptoms, limited insight**  GP: “If they’re in either a state of denial or have limited insight, it might be more difficult because you’re not really pushing against an open door...It’s a difficult one because, I mean, in a way we could always insist that people were seen and say ‘Look, you know, I think it’s really, really important’. I mean, the sort of, extreme of that would potentially be being a form of bullying because you can, you can literally say ‘oh, I really do think you should see them and I don’t care what you think, because I think you’re dementing and you have to be seen and making the diagnosis is very important for you’ which, you know, actually, sounds massively paternalistic and it is...” (Dhedhi et al., 2014)  GP: ‘‘They partially block off. They contradict and claim: ‘No, that’s not true; I’m not soft in the head. I’m not insane.’ and they feel as if I had accused them’’. (Kaduszkiewicz et al., 2008)  **Reluctant to accept or not wanting to know the diagnosis**  GP: “There are major issues from the patient perspective in that the vast majority of people...want to know if they've got cancer but there's not many people that will want the same with dementia.” (Crombie et al., 2024)  PN: “one of the biggest barriers is patient acceptance” (Gibson et al., 2021) |
| The patient's situation and context | | **The patient’s wish, need and right (not) to know**  GP: “’Before we go any further, I just want to make it clear that I don’t want you tell me that I’ve got Alzheimer’s’ […] I was sure after two consultations that she was able to make her own decision about whether or not she wanted to pursue being investigated further. She went a little way, but at this point she elected not to take it any further.” (Dhedhi et al., 2014)  **Diagnosing dementia is related to the patient’s context**  GP: “Rural patients are different than urban patients. [...] Rural patients typically don’t want to have to go into a city, they don’t want to have to live in an apartment [...]. It’s a tougher diagnosis to give an 85-year-old farmer than an 85-year-old executive guy. [...]. You don’t just take the guy away from his farm.” (Constantinescu et al., 2018)  GP: “You are not always dealing with just dementia, you are often dealing with elderly frail people so I don’t suppose the dementia diagnosis as such would make much difference . I would usually see dementia in a spectrum of conditions that an elderly person might have.” (Hansen et al., 2008) |
| Fear of dementia | | **Fear of the diagnosis**  GP: "Older people avoid talking about dementia. They get scared when they hear about dementia or you give them a dementia screening. Even if I feel that older people  have cognitive problems, they will deny it, reject it, and be reluctant to admit it. The patient’s and the family’s awareness are not strong enough, and many things (dis- ease symptoms) are covertly ignored as a result. How do I continue?" (Gong et al., 2023)  PN: “they still see it as a real fear” (Gibson et al., 2021)  **Diagnosis is emotionally difficult**  GP: “. . . I think it’s also very important not to scare people witless in maybe what could be the very early stages of dementia. . . and you don’t want to plunge someone into a depression, they are going to go completely mad in six months, you have to be very sensitive to how someone  is going to take any information. . . .” (Moore & Cahill, 2013) |
| The availability and attitude of family members | | **History provided by family is essential information**  GP: “I think people who are more socially isolated . . . may don’t have family around or a lot of friends . . . they might not come to anybody else’s attention because they manage somehow to function.” (Bature et al., 2018)  **Family members usually report the symptoms**  GP: “‘...it is often the family that notices the small changes and so more often than not, they are the ones who will bring them in.” (Chithiramohan et al., 2019)  **Social support system influences decision-making**  GP: “In general, hospitalization isn’t always related to the progression of the disease itself, it’s the family’s level of tolerance, not the disease, you see. They tell me ‘I can’t do this any more, it’s too hard.’ That’s what it is most of the time.” “It’s not so much on the medical front that there’s a problem. I think it’s more often purely on the social level.” (Lahjibi-Paulet et al., 2012) |
| CLUSTER 3: INDIVIDUAL DETERMINANTS OF PRIMARY CARE PRACTITIONERS | | |
| Factors undermining PCPs’ confidence | **Complex to interpret results of cognitive tests**  GP: “So this might be somebody who is well educated, they score well on their mini mental state exam (sic), but I’m thinking, well look is that just because they have a high level of education? Or is it that they’re cognitively OK?” (Foley et al., 2017)  GP: “Sometimes it’s dubious, when a person can seem quite confused and still perform quite well on these tests. So, it’s not always totally clear, or easy, to make a diagnosis. When it hasn’t progressed that far.” (Lindeberg et al., 2022)  **Contradictions between different information sources**  GP: “There’s often denial and some verbal discussions when you see a person with dementia and a relative together. Where they have totally different views on what issues there are.” (Lindeberg et al., 2022)  **Lack of knowledge, training or education**  PN: “but I don’t know what to do with these people. The poor carer comes in and they’re nearly crying and pulling their hair out ... but you still feel that you [are] useless because you don’t know what to do for them” (Gibson et al., 2021) | |
| Factors contributing to PCPs’ confidence | **PCP feels comfortable conducting cognitive screening and making the diagnosis**  GP: “I get the impression that what I actually have available in terms of tests, which take me 10 minutes, plus these people’s living situation, is enough to make a diagnosis. In other words, it seems that every time I referred someone thinking that it was Alzheimer’s, it was.” (Lahjibi-Paulet et al., 2012) | |
| PCPs’ perceptions of their role | **The role of the PCP is recognizing cognitive problems and initiating or conducting the diagnostic work-up**  GP: “Picking up the signals ... and then deciding if you can diagnose and advise that person yourself or if you need to refer.” (Prins et al., 2016)  **PCPs believe that a diagnosis should be made or confirmed by a specialist**  GP: "We can’t diagnose without qualification. How can we talk to them about dementia? Of course, they should see a specialist to confirm the diagnosis." (Gong et al., 2023)  **PCPs have a unique position to act as a guide**  PCP: “I think trust is a big thing...dementia is a scary thing for patients. Having that core of trust in the relationship for established patients and being able to as a provider guide patients and caregivers through that...that’s golden....To be able to leverage that in the care of patients with dementia...[i]t’s a privilege...a real service that you can provide your patients.” (Bernstein Sideman et al., 2022)  **The practice nurse may have an important role in identifying and discussing dementia**  PN: “But by gaining that therapeutic trust building up, that relationship with them. Having that extra time to listen to them ramble on a little bit. That’s where we’re probably going to get a better picture. Also having that little bit of extra time with them allows us visually to see how they behave, how they inter- act. How their thought processes work instead of just perhaps putting them on the spot in that brief GP type setting” (Gibson et al., 2021) | |
| PCPs’ perceptions of the aim of the diagnostic process | **Meeting patients’ and family members’ needs and preferences**  GP: “You can ask people that: ‘Oh what makes you come here now, what do you expect from me?’ I can think of all sorts of things, but before I get started right away: ‘I [...] am just curious  what you would like me to do. And when will you leave satisfied?’ [...] If people come  themselves, it is rare for dementia to occur. When people come themselves, it is usually fear of dementia.” (Tromp et al., 2021)  **Prioritizing well-being and safety over confirming a diagnosis**  GP: “I look after you, you are my concern and less of a concern is which label I use for what you have” (Dhedhi et al., 2014)  **Supporting to live independently and to stay at home**  PCP: “We know our people... ...Yeah. So it's just building that bridge and connection and making them feel safe and trust and helping them to be aware and know of these illnesses, that we are there to help them, not to lock them away. Yeah.” (Bryant et al., 2021)  GP: “You give them some lead-in comments … that perhaps they’ve noticed that they’ve been having some difficulties with their memory. So, you’re actually allowing them an opportunity to recognize the problem and discuss it … in a non-threatening way and it also has to offer them some hope … “You have some memory impairment. I mean, but you’re still functioning and living independently and my role is to try and maintain that for as long as possible” … It’s a good opportunity to be able to reassure patients that there’s strategies and things that we can do to maintain their independence … I will often encourage patients to bring in their spouses or their children … it just keeps the lines of communication open …[and reinforces] any decisions that have been made together … I would never support a relative … wanting to hide the information from a patient. I don’t think that’s anybody’s role to do.” (Phillips et al., 2012) | |
| CLUSTER 4: EXPECTATIONS OF PRIMARY CARE PRACTITIONERS REGARDING THE CONSEQUENCES OF A DIAGNOSIS | | |
| Positive (expected) outcomes of diagnosis | | **Improving well-being and quality of life**  GP: “You don’t have to cure it but at least you could reduce their sufferings and you could improve their quality of life certainly, there are ways you could do to help them. If you don’t have that mind-set, then that could act as a real barrier because the diagnosis just gets delayed and delayed and before you know it they are no longer in the early stages.” (Chithiramohan et al., 2019)  **Access to care and additional support**  PCP: “Patients having that diagnosis open the door up to other services that we can get, whether it be home health care, certain in-home devices, some medication administrative assistance. Even long-term care in extended-care facilities.” (Abe et al., 2021)  **Future planning and the possibility for patients to decide for themselves**  FP: “[Early diagnosis] helps the individual and family to make plans. [I]f you pick up the dementia early enough, they get [personal directives] while they can, before things go south.” (Constantinescu et al., 2018)  **Counselling or guidance**  GP: “‘Well I think the most important thing is to explain to the patient what’s going on with them so the patient is aware of the fact that you believe there’s a process taking place in their blame that is the reason why they’re having the symptoms they’re having, and explaining that to their family as well, so then you can treat them most effectively to manage the various aspects of the dementia.” (Steiner et al., 2020)  **Medication can be used in suitable cases**  GP: “Early diagnosis of dementia is very important. Early diagnosis and timely treatment can slow down the disease process” (Huynh-Truong et al., 2023) |
| Limited added value (expected) of diagnosis | | **No clear effect of treatment**  PCP: “I have very mixed feelings about even starting people on the medications because I feel like the evidence for them that they are effective is pretty limited.” (Abe et al., 2021)  PCP: ”Yeah, because it's not treatable so it's a terminal condition, for want of a better description. I tend to find that's usually the biggest barrier” (Bryant et al., 2021)  GP: “In my opinion, [there is] no real effective treatment for it [dementia]. And so making the diagnosis is almost like saying to somebody, yes you have this problem, and we are going to help you with this problem, but we can’t really do very much for it.” (Chithiramohan et al., 2019)  **No value in early diagnosis**  GP: “So then I think, yes then you can impose that on someone and say: ‘Look, you have a dementia label.’ But it must have a function, or it must be able to contribute to a better happiness, so to speak.” (Tromp et al., 2021)  **Limited sources of care**  GP: “For me, all these statistics don’t mean a thing, since nothing is provided for these people... It’s nothing but marketing... well, not exactly, but we’re in some sort of advertising blitz that does nothing but add pressure, but on what since there’s nothing, no resources, nothing. For me, it’s all show.” (Lahjibi-Paulet et al., 2012) |
| Negative (expected) outcomes of diagnosis | | **Diagnosis causes emotional distress**  FP: “On the other hand, making a diagnosis can be emotionally difficult for patients and their families, thereby hindering the process of dementia care.” (Constantinescu et al., 2018)  GP: ”… sometimes I have to make a judgement knowing the patient … where you’re just far better not to tell them … as soon as you[do] …they go downhill.” (Phillips et al., 2012)  **Driving licence must be taken away**  **FP:** “You know that now the driver’s license is going to have to be taken away and you know that a lot of these people have to move from where they’re living somewhere else at some point, families get torn apart.” (Constantinescu et al., 2018)  **Not wanting to medicalize the aging process**  GP: “I’m interested in trying to let people live a normal life and not become a patient. The problem is that we are medicalizing the ageing process..” (Hansen et al., 2008)  GP: “In my opinion, we are talking about an aging condition. So we make... we have developed a lot as doctors that make people get older and I think Alzheimer’s really is an aging condition that of course involves all kinds of things in our society, but ultimately because people get old enough. That’s just like those people with a prostate [cancer], if you grow old enough then you die with Alzheimer’s.[ . . . ] Yes, I think that indeed makes my opinion different from someone who sees it as a disease [...] yes I mean Alzheimer’s is a part predisposition, a part environment, part aging, there are a lot of things.” (Tromp et al., 2021) |
| CLUSTER 5: FACTORS RELATED TO THE HEALTHCARE SYSTEM | | |
| Failures of dementia policy | **Time-constraints in general practice**  GP: “Yeah, yeah, remember how the NHS works, a GP practitioner works ten minute appointments so I have ten minutes to see one patient for one problem, now so the way you diagnose is within the resources you have and a time limit you have; you might see the patient probably 6 or 7 times before you start thinking that this is a firm diagnosis of AD.” (Bature et al., 2018)  **Dementia is a low priority health condition**  PCP: “I think it's easy to just gloss over memory issues and certainly, clients at [service] present with a whole range of complex issues and usually competing priorities. I think memory is probably one of those things that are put to the bottom of the list and we often don't get to it, whether that's consciously or subconsciously. I think it's usually more pressing needs until the family presents in crisis sometimes.” (Bryant et al., 2021)  **Lack of care available**  GP: “There is also not enough capacity at the receiving end to deal with all the diagnoses coming through that we make. So we make the diagnosis, fine. There is a memory clinic. Fine. But there aren’t enough people there to deal with the bulk of referrals and to get the diagnosis sorted.” (Chithiramohan et al., 2019)  GP: “Because a barrier that you’d have afterwards is that once they have reached the diagnosis, there isn’t really much support afterwards for them . . . The family are left with this diagnosis when it comes to afterwards . . . the aftercare there is not really much until things go really, really bad.” (Chithiramohan et al., 2019)  **Lack of funding for dementia screening, diagnosis and services**  GP: “This is a loss-making business for a general practitioner. In its current state, it is simply an economic ball and chain.” (Wangler & Jansky, 2020) | |
| Insufficient guidelines and tools | **No clear guidelines or referral mechanisms**  GP: "(referral) vague concept. The pathway is unclear as to where exactly to go and what to do next for the positive patients (who screen out)." (Gong et al., 2023)  GP: “If we could be supported by guidelines and checklists, I think we would feel somewhat stronger and more confident, and we would [more often] not refer.” (Prins et al., 2016)  **Need for availability of an accepted, valid and practical tool for PCPs**  GP: “Well, I would be happy if we could have an accepted, valid instrument and then we could make the diagnosis ourselves. I certainly feel, when we think about cost saving and fewer referrals to secondary care, that it is really useful if we were to do this.” (Prins et al., 2016)  GP: “Well, I think it’s nice when you can do it yourself, but because it is an essential diagnosis, I think you really need reliable diagnostic tests. And I think that takes a lot of time.” (Prins et al., 2016)  **Diagnostic tools provide insufficient guidance**  GP: “Oh sometimes I do an MMSE. ... I don’t find it that useful. I mean ... from conversation you can really find which way they’re going. I mean... so the MMSE is ... it doesn’t give me additional information I find.” (Murphy et al., 2014)  GP: “I think the downside of the MMSE is that scores stay high for quite a long time, even though you notice that someone has really changed.” (Prins et al., 2016) | |
| Advantages of interprofessional collaboration | **Advantages of practice nurse role: extra time, home visit, and less threatening nurse-patient relationship**  PN: “But by gaining that therapeutic trust building up, that relationship with them. Having that extra time to listen to them ramble on a little bit. That’s where we’re probably going to get a better picture. Also having that little bit of extra time with them allows us visually to see how they behave, how they interact. How their thought processes work instead of just perhaps putting them on the spot in that brief GP type setting” (Gibson et al., 2021)  PN: “the GPs feel that their relationship can be threatened if they broach the subject ...we have the gift of time ... we can actually build rapport with the patient and speak to them” (Gibson et al., 2021)  **Strong team culture and interprofessional collaboration facilitates diagnosis**  GP: “Together we can offer a much better diagnostic work-up at home and see what goes wrong in the home situation, than at a specialised memory clinic. At home you observe so much more and this is so valuable. And as a GP or practice-based nurse specialist, you have much easier access and for the patient it’s less threatening. The patient and their family often give back that they find this a very welcome way for a diagnostic work-up.” (Vissenberg et al., 2018)  **Practice nurses are not always supported as proactive healthcare practitioners**  PN: “The thing is, we’ve got - we’re limited to what we can do. We can suggest things, but we’ve sort of - you’ve got to bring it to the GP’s attention sort of thing if it’s a patient. It’s - we can’t just go ahead and order things like that. It’s got to come from the GP, so we’re sort of...We can plant the seed, we plant the seed pretty much and say, you know, we’re just a bit concerned about this patient such-and-such, or what- ever, and then they – whatever” (Gibson et al., 2021) | |
| CLUSTER 6: SOCIETAL FACTORS | | |
| Stigma of dementia | | **Avoidance of the word ‘dementia’ or Alzheimer’s’ in communities**  PCP: “They'll just say it's about memory problems or they're not the same as they were before. I don't hear the word dementia being used a lot by people...” (Bryant et al., 2021)  PCP: “It's not necessarily labelled in that sort of language if you get me.... It's just oh so and so's getting a bit old or that they're just forgetting things a bit.... I suppose that you could say that the symptoms are recognised and supported in community, but in terms of actually recognising that dementia or that could be dementia, that that hasn't been - it hasn't really been talked about like that.” (Bryant et al., 2021)  **Dementia is perceived as a stigma within society**  GP: “Unfortunately there is still stigma, and a lot of people think that rather than having different degrees of dementia, it means that you are doolally, and that you have lost your marbles, you are incontinent and go into a nursing home. So that sort of association just delays the patient or family coming in or just plain denial.” (Cahill et al., 2008)  **Negative attitudes towards people with dementia among HCP’s**  PN: “this patient’s saying, no, I’m all fine. I’m thinking, you’re as demented as anything” (Gibson et al., 2021) |
| Cultural and language barriers | | **Challenges in assessment due to language barriers, illiteracy, and non-applicable diagnostic tools**  GP: “Literacy is another problem especially with the older generation. So, I find it difficult to know what tools to use to diagnose a dementia […] you use your MMSE and it will ask you like when was World War One and they just won’t know, not because . . . it’s because they never knew in the first place. So I don’t think it is tailored to different languages and cultures . . . things like there is the GPCOG, the 6IT, and the MMSE and all of those worked well with the English-speaking population. But I think that the population that we are in . . . it doesn’t transfer very well.” (Chithiramohan et al., 2019)  “A culturally sensitive diagnostic cognitive screening instrument would then be a good tool to make things more visible and clear” (Vissenberg et al., 2018)  **Dementia is considered a taboo or bad karma**  GP: “Either it (the response) can be a form of belittling in a way, or they relate it to Gods will ... and then we are not meant to do anything about it.” (Sagbakken et al., 2018) |
| Lack of knowledge and awareness | | **Limited awareness and understanding about dementia**  PCP: “There isn't self-recognition and there certainly is less family recognition that there may be an issue that is potentially treatable or requires extra services.” (Bryant et al., 2021)  **The belief that memory problems are part of ageing**  GP: “‘High tolerance from patients and relatives of the vagaries of old age.” (Cahill et al., 2008)  GP: “Yes I’m forgetting a few things here and there but this is to be expected isn’t it? I am 80 after all!’’’ And that sort of mindset is only going to delay early diagnosis of dementia.” (Chithiramohan et al., 2019)  GP: “…not that they are covering but they feel it is normal: he is old, he is forgetting. They don’t realise that he is going to be dangerous. Those sort of things. So these are the barriers we have.” (Chithiramohan et al., 2019) |
| CLUSTER 7: STRATEGIES AND ACTIONS OF PRIMARY CARE PRACTITIONERS | | |
| Dealing with the challenge of sensitivity | | **PCPs avoid the burden of delivering bad news by referring to the specialist**  GP: “I send them to a specialist on purpose. Announcing the diagnosis is precisely the moment when the presence of several people is necessary. It’s too violent for one person to tell another. I think it’s a good idea.” (Lahjibi-Paulet et al., 2012)  GP: “If someone else could do it [disclose the diagnosis], I, I’d put it back … put the blame on them you know …I think it makes it a little easier. I can then be supportive rather than sort of knocking the socks off them.” (Phillips et al., 2012)  GP: “Let a specialist deliver the bad news … I usually utilise my colleagues as a fall-guy to actually present the diagnosis.” (Phillips et al., 2012)  **PCPs communicate that the patient is in charge during the process**  GP: “Usually I tell them ‘It is not that we will decide everything for you, you always remain in charge’” (Linden et al., 2024)  **PCPs search for the 'right' moment**  GP: ”As a general practitioner, it is in your interest for the patient to be in a psychologically and socially stable state. Therefore, in some cases, you accept situations even when you know it is very likely that there is an underlying issue. You begin to wonder when is the correct time to present a patient with such a negative perspective—telling them that they have been diagnosed with dementia. [...] There is a danger that, as a physician, you end up in a downward spiral of inaction.” (Wangler & Jansky, 2020)  **Taking it slowly and gradually introducing the topic**  GP: “I attempt, beforehand, to say something along the lines of, I may ask a few silly questions, to warn the patients, and just wanted to know how good your memory is, and so not to offend them. I wouldn’t say that the nature of the tools would stop me using them.” (Chithiramohan et al., 2019)  GP: “One of the other problems is that people often, in this situation, particularly somebody who has managed extremely well, is very reluctant to have any support, very reluctant to uhm see themselves as giving up any of their independence. Uhm, so it’s uhm, I think what’s really important is to be very clear, to take it really quite slowly and to make sure that the patient understands that you’re going along several tracks at once. You know, let’s check that you’re not anaemic, that you haven’t got a thyroid problem, you know, those kinds of things. And, certainly, I would never, in these kinds of circumstances suggest a visit from the memory clinic people at the first stage, because you really have to work at that a little bit.” (Dhedhi et al., 2014)  GP: “. . . I think it’s also very important not to scare people witless in maybe what could be the very early stages of dementia. . . and you don’t want to plunge someone into a depression, they are going to go completely mad in six months, you have to be very sensitive to how someone is going to take any information. . . .” (Moore & Cahill, 2013) |
| Sensitivity causes hesitation among PCPs | | **Hesitancy to label the patient**  GP: “I think that the label is the problem. I think all of us (GPs) are reluctant to label somebody because that immediately puts them in a category of high dependency . . . so I think all of us . . . you know we’ll muddle along in a grey area until . . . it’s clear a patient can’t manage on their own . . .” (Cahill et al., 2008)  **Wait and see approach until problems become evident**  GP: “Very often you know these patients very very well and have seen them over many years . . . and maybe you don’t notice, because of your lack of memory . . . their lack of memory and then it’s really only when a crisis occurs, you know, something happens that sort of makes everybody stand back and say oh my God its really obvious” (Cahill et al., 2008)  GP: ‘‘I also think that non-disclosure is all right as long as the patient doesn’t suffer and is in some way happy and nothing bad happens.’’ (Kaduszkiewicz et al., 2008)  GP “When I think: this might give problems with medication and all that, I’ll do something.” (Prins et al., 2016)  **Fear of ruining the doctor-patient relationship**  GP: ‘‘I avoid conflict with the patient. I don’t want him to get angry and I don’t want to lose him as my patient. It’s not for financial reasons; every patient I lose will be replaced by another, but for me mutual trust is very important. And I think that such a disclosure can be very offending for the patient’’. (Kaduszkiewicz et al., 2008) |
| PCPs try to weigh up dilemmas | | **Weighing up benefits and negative consequences of a diagnosis**  GP: “I think it’s hard to know the initial thing of how much to inform the patient. Sometimes you do get the impression that the patient is better off not having a formal diagnosis... But as long as the patient is benefiting from that, that their care is going to be maximized” (Foley et al., 2017)  **Balancing patient autonomy and preventing risks or crises**  GP: “And, as I recall, she really wasn’t very keen for anybody to come in initially and there was no reason for me to consider that she was highly at risk and so, you know, in those circumstances, if somebody is basically refusing referral, unless they haven’t got competence, you know, you just have to patiently wait...” (Dhedhi et al., 2014)  **Balancing patient autonomy and preventing risks or crises &**  **Negotiating conflicting priorities and expectations of involved ones**  GP: “I think it is a negotiation as to what one can do. So, you can always negotiate harder and I certainly could have negotiated harder [in this particular case] but I would prefer—I mean, maybe it’s a personal style—I certainly would prefer that, you know, they come, or eventually come round to your view. Now, the catch with that is that sometimes what happens is you get a crisis. You could say ‘Well, you could have intervened earlier’. Yeah! But that then would have been counter to providing him with any particular form of, you know, autonomy. So, that’s a constant struggle, just knowing, ‘could I have done that?’ ...it’s a constant struggle. I mean, it’s difficult to know, because how would I know anyway whether it was a better or worse decision?” (Dhedhi et al., 2014) |
| Approaches to start the conversation | | **Proactive approach, routine health check, or screening prompts conversation about dementia**  GP: “I’m starting to get from actually our Medicare Annual Wellness Visit Form, which has a question that will say something about memory, and they’ll circle, “Yes,” just really not thinking anything of it. And then we start to have that conversation.” (Abe et al., 2021)  **Having an opening to address concerns**  GP: “I already had an opening: ‘You know that your son has phoned me, he’s a little bit concerned.” (Dhedhi et al., 2014)  **Continuity of care enables recognition and initiation of the conversation**  GP: “Uhm and so it’s much easier, it’s much easier to have that conversation, it’s also much easier to have it when you have known somebody over a very long time.” (Dhedhi et al., 2014)  **Holistic approach supports looking further than the known diseases**  PN: “It isn’t all just about facts and figures. It’s not about the one disease or the one comorbidity, it’s the whole lot impacting on each other ... you can’t isolate one disease from the other. You’ve got to - if someone’s got dementia and diabetes, for example, you can’t just treat the diabetes without having stuff in place for the dementia and likewise” (Gibson et al., 2021) |
